# Reducing the number of systematic biopsy cores in the era of MRI targeted biopsy—implications on clinically-significant prostate cancer detection and relevance to focal therapy planning

**DOI:** 10.1038/s41391-021-00485-3

**Published:** 2022-01-14

**Authors:** Alvin Y. M. Lee, Kenneth Chen, Yu Guang Tan, Han Jie Lee, Vipatsorn Shutchaidat, Stephanie Fook-Chong, Christopher W. S. Cheng, Henry S. S. Ho, John S. P. Yuen, Nye Thane Ngo, Yan Mee Law, Kae Jack Tay

**Affiliations:** 1 Department of Urology, Singapore General Hospital, Singapore, Singapore; 2 Health Services Research Unit, Division of Medicine, Singapore General Hospital, Singapore, Singapore; 3 Department of Urology, Sengkang General Hospital, Singapore, Singapore; 4 Department of Pathology, Singapore General Hospital, Singapore, Singapore; 5 Department of Radiology, Singapore General Hospital, Singapore, Singapore

**Keywords:** Outcomes research, Prostate cancer

## Abstract

**Background:**

The optimal number of systematic biopsy cores in the era of multi-parametric MRI targeted biopsy remains unclear, especially on its impact of focal therapy planning. Our objective is to investigate the impact of reducing the number of systematic cores on prostate cancer detection in the era of MRI-US fusion targeted biopsy and as well as its relevance in template planning for focal therapy.

**Materials and methods:**

A retrospective analysis of 398 consecutive men who underwent both systematic saturation (~24 cores) and MRI-US fusion targeted biopsy was performed. Four reduced-core systematic biopsy strategies (two-thirds, half, one-third and one-quarter systematic cores) were modelled and the detection rates of clinically-significant prostate cancer (csPCa defined as grade group ≥2) were compared to that of a full systematic biopsy using McNemar’s test. Focal therapy treatment plans were made based on positive cores on combined (targeted and systematic) biopsy and the various reduced-cores strategies to compare the proportion who had a change in treatment plan.

**Results:**

csPCa was detected in 42% (168/398) of this patient cohort. Non-targeted systematic saturation biopsy had a 21% (83/398) csPCa detection rate. Our four strategies reduced the mean number of non-targeted systematic cores from 21.8 to 14.5, 10.9, 7.3 and 5.4 cores and their csPCa detection rates were significantly decreased to 16%, 13%, 9% and 8% respectively (all *p* < 0.05). Compared to the reduced-core strategies, a full systematic saturation biopsy resulted in change to the focal therapy treatment plan in 12% (2/3 cores), 19% (1/2 cores), 24% (1/3 cores) and 29% (1/4 cores) of the time (*p* = 0.0434).

**Conclusions:**

Reducing the number of systematic biopsies when performing an MRI-targeted biopsy leads to reduced detection of csPCa and alter the treatment plans for focal therapy, possibly limiting its oncological efficacy.

## Introduction

With multi-parametric magnetic resonance imaging (mpMRI), targeted fusion biopsy has been shown to better detect clinically-significant prostate cancer (csPCa) compared to transrectal biopsy [1]. However, systematic biopsy cannot be omitted as 5–16% of csPCa may be missed [1–4]. To investigators interested in focal therapy, systematic biopsy is an essential step to exclude csPCa in mpMRI-negative areas and to ensure that the patient truly has a discrete lesion suitable for focal therapy [5]. As systematic biopsies might overlap into MRI suspicious areas, the true utility of these systematic biopsies that only include non-targeted areas remain unclear [6]. Our previous study showed that non-targeted systematic biopsy had a csPCa detection rate of 21% and found that omitting systematic biopsy (excluding overlapping cores into the MRI lesion) would only result in only 3% having csPCa missed [7]. Prostate cancer is known to be multi-focal and systematic biopsy serves to identify foci out of the index lesion which may not be well-visualised on MRI. To date, the critical clinical question of the optimal number of systematic biopsy cores remains an unanswered one, especially in the context of treatment planning for focal therapy [5].

Through modelling systematic biopsies with a reduced number of cores, the primary aim of our study was to determine if reducing the number of cores on systematic biopsy would significantly affect the csPCa detection rates. To understand its clinical impact, our secondary aim was to analyse how the reduced-core strategies would impact the focal therapy template planning.

## Materials and methods

### Patient cohort

This study was a retrospective analysis of patients with any suspicious lesion on mpMRI who underwent both systematic and MRI-US fusion targeted biopsy using our proprietary iSR’obot Mona Lisa^TM^ transperineal prostate biopsy platform (Biobot Surgical, Singapore) between January 2015 to January 2019 at a single centre. This study was approved by our Institutional Review Board (2009–1053-D). Our mpMRI protocol and biopsy technique were previously described [8–10].

### Multi-parametric MRI protocol

Patients underwent 3-Tesla MRI in accordance to current international MRI prostate guidelines [11]. Majority of the mpMRI images were read by a senior radiologist (Y.M.L.) with 8 years of experience in prostate MRI with the rest being read by radiologists with at least 4 years of experience. All suspicious lesions were assigned a Prostate Imaging Reporting and Data System Version 2 (PI-RADS^TM^ v2) score [12]. The prostate outline and suspicious lesions were marked by the radiologists using our fusion software to produce a 3D MRI model (Urofusion^TM^, Biobot Surgical, Singapore).

### Prostate biopsy

All biopsies were performed under general anaesthesia. The pre-biopsy mpMRI 3D model of the prostate is fused to the intraoperative transrectal US 3D prostate model using a non-rigid software fusion algorithm (Urofusion^TM^, Biobot Surgical, Singapore). The systematic saturation and targeted biopsy are then automatically planned using the in-built, volume-dependent computer algorithm with on-table operator optimisation. The systematic saturation biopsy protocol is uniform and planned to cover all areas of the prostate (transitional, peripheral, anterior fibromuscular stroma) from base to apex, excluding the urethra and seminal vesicles. The systematic saturation cores are planned independently of the lesions found on MRI and each core can be adjusted, added or removed by the operator to avoid the urethra and/or achieve adequate systemic coverage. The targeted cores are taken first, followed by the systematic cores during the same transperineal procedure. International Society of Urological Pathology (ISUP) grade group ≥ 2 cancers were considered to be csPCa [13].

### Biopsy map review

As the systematic saturation cores were planned independently of the MRI lesions, some systematic cores were mapped within the MRI regions of interest and were labelled as such. We reviewed the biopsy maps of each patient to reclassify systematic cores which sampled the MRI regions of interest as targeted cores instead. In this study, systematic biopsy only included systematic cores that were not within the lesions identified on MRI. Overlapping systematic cores into the target zone were reclassified as targeted cores instead, as they merely resample the target lesions and do not provide information on cancer detection outside of the MRI target zone.

### Modelling for reducing the number of systematic cores

Since every systematic biopsy core is deployed in a standard fashion, we modelled four reduced-core systematic biopsy strategies, in order of decreasing number of cores (Supplementary Table 1). The detection rate of each strategy is then calculated by taking the mean detection rate of all the models within the strategy.

### Focal therapy treatment planning

**Fig. 1:**
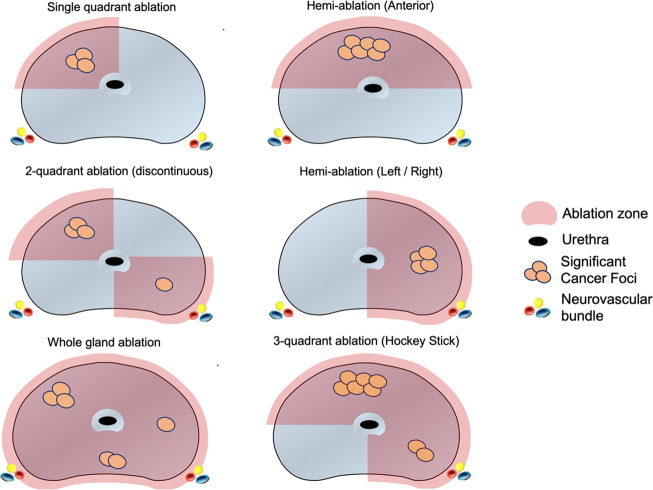
Schematic showing various focal therapy templates that were used to treat clinically-significant cancers. Focal therapy treatment plans were made based on the location of clinically-significant cancer detected using each reduced-core strategy. These plans were compared to targeted biopsy only and a combined targeted and full systematic saturation biopsy to see if there were any changes in treatment plan. The proportion of men who had a change in treatment plan were then compared across each reduced core strategy.

Those suitable for focal ablation had templates mapped out by an experienced practitioner of focal therapy (KJT) and the ablation templates were classified as single quadrant ablation, hemi-ablation (anterior or lateral), three-quadrant ablation or whole gland ablation (Fig. 1). Patients who had bilateral posterior quadrant involvement were not considered candidates for focal therapy as the impairment to sexual function was deemed to be as high as that of radical therapy. Patients were generally selected using criteria adapted from recommendations from an international Delphi consensus – PSA ≤ 10 ng/mL, single MRI lesion with volume less than 1.5 mL with grade group 2 or 3 on targeted biopsy and a prostate volume of less than 50 mL [5]. Patients who had peri-urethral disease or had high-risk disease (prognostic grade group 4 and above) were deemed not suitable for focal therapy and hence, were recommended for whole-gland radical therapy (e.g. radical prostatectomy or radiotherapy). If only grade group 1 or benign histology were found in each strategy, active surveillance (conservative therapy) would be recommended as a treatment strategy for purposes of this study. Focal therapy treatment plans were first designed separately based on foci of csPCa identified on: (1) targeted biopsy alone, (2) combined targeted and full systematic biopsy and (3) combined targeted and each reduced-core systematic biopsy strategy. Following which, the change in treatment plan (defined as a change in ablation quadrants or complete change of strategy to whole-gland radical therapy) were compared. The average proportion among all the models within each strategy was taken to be representative of the proportion of those who had a change in treatment strategy for each strategy.

### Statistical analysis

The csPCa detection rate of each reduced-core strategy is compared to that of the complete systematic biopsy using McNemar’s test. The proportion of men who had a change in treatment plan for the various models were compared using Chi-square test. A *p* value of < 0.05 was considered statistically significant.

## Results

### Baseline characteristics and detection rate of prostate cancer

The baseline characteristics of the 398 men included in our study are displayed in Table 1. Prostate cancer was detected in 54% (213/398) while clinically significant prostate cancer was detected in 42% (168/398). The detection rate of csPCa was 21% (83/398) on systematic biopsy and 39% (155/398) on targeted biopsy. Using combined targeted and systematic biopsy, csPCa detection rates for PI-RADS 3, 4 and 5 were 13%, 35% and 83% respectively. Seventy patients (18%) had csPCa detected on both targeted and non-targeted systematic biopsy, 13 men (3%) had csPCa detected exclusively on non-targeted systematic biopsy and 85 men (21%) had csPCa detected exclusively on targeted biopsy.

**Table 1:** Baseline characteristics of men with csPCa outside of the MRI lesion detected on systematic biopsy.

Baseline characteristic	Total	No csPCa	csPCa detected outside of MRI lesion	*p* value
Patients, *n* (%)	398	315 (79)	83 (21)	
Race, *n* (%)				
Chinese	347 (87)	277 (88)	70 (84)	
Non-Chinese	51 (13)	38 (12)	13 (16)	0.383
Mean Age, years (±SD)	65.7 (±7.8)	64.9 (±7.7)	68.6 (±7.4)	<0.001
Prior negative biopsy, *n* (%)	133 (33)	122 (39)	11 (13)	<0.001
Mean prebiopsy PSA, ng/mL (±SD) (*n* = 393)	10.1 (±8.4)	9.6 (±7.2)	12.2 (±10.7)	0.045
Mean MRI-US fusion prostate volume, mL (±SD)	43.5 (±18.6)	45.3 (±18.2)	36.7 (±18.5)	<0.001
Mean PSAD	0.27 (±0.25)	0.24 (±0.22)	0.38 (±0.33)	<0.001
Median number of MRI lesions (IQR)	2 (1–2)	2 (1–2)	2 (1–3)	0.093
PI-RADS score				
3 (%)	69 (18)	68 (22)	1 (1)	
4 (%)	240 (60)	191 (61)	49 (59)	
5 (%)	89 (22)	56 (17)	33 (40)	<0.001
Median number of cores, *n* (IQR)				
Total	33 (28–39)	33 (28–39)	32 (26–36)	0.038
Systematic	23 (19–29)	24 (20–29)	21 (18–26)	0.004
Targeted	9 (6–12)	9 (6–12)	9 (7–12)	0.105
Median number of overlap cores, *n* (IQR)	5 (4–7)	5 (4–7)	5 (4–7)	0.408

*GG* Grade Group, *PSAD* prostate specific antigen density, *PI-RADS* Prostate Imaging—Reporting and Data System.

### Detection rates of csPCa using reduced-core strategies

Strategy (1) to (4) involved reducing the mean number of non-targeted systematic cores from 21.8 to 14.5, 10.9, 7.3 and 5.4 cores respectively (Table 2). The mean clinically-significant cancer detection rate of reduced-core strategy (1), (2), (3) and (4) were 16%, 13%, 9% and 8% respectively which was significantly lower than the 21% detection rate of a full systematic biopsy (all p < 0.001, Table 2). In men who had prior negative biopsy, only strategy (3) and (4) resulted in a significant decrease in csPCa detection rate on systematic biopsy (4 and 4% vs 8% respectively, both *p* = 0.031). No significant difference in csPCa detection rate was observed when strategy (1) and (2) were compared to full systematic biopsy in men who had a prior negative biopsy (*p* > 0.05).

**Table 2:**
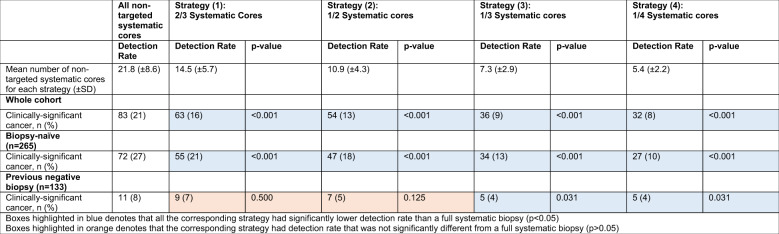
Mean detection rate of prostate cancer using strategies involving reduced number of non-targeted systematic cores.

### Distribution of recommended treatment therapies

The distribution of the recommended treatment options is displayed in Supplementary Table 2. Using combined targeted and full systematic biopsy, 42% (*n* = 32) were deemed not suitable for focal therapy and were recommended for whole-gland radical therapy. The main reasons were peri-urethral gland involvement (*n* = 28) and high-risk disease such as grade group>/= 4 or extensive disease (*n* = 4).

### Changes in focal therapy treatment plan

When compared to the treatment plan based on targeted biopsy alone, the addition of a full systematic biopsy resulted in a change in treatment plan in 44% of men, with 10% of patients deemed unsuitable for focal therapy (Table 3) The proportion of men who required a change in treatment plan decreased as a less thorough systematic biopsy was performed (i.e. decreasing number of systematic cores from strategy 1 to 4) from 44 to 16% (*p* < 0.001). Of those who had a change in treatment plan, the majority was due to an increase in focal therapy ablation zone.

**Table 3:** Proportion of men who had changed in treatment plan when compared to treatment plan based on targeted biopsy only.

	All cores (Targeted + all systematic cores)	Strategy (1): Targeted + 2/3 Systematic Cores	Strategy (2): Targeted + 1/2 Systematic cores	Strategy (3): Targeted + 1/3 Systematic cores	Strategy (4): Targeted + 1/4 Systematic cores	
No change in focal therapy template/no change in decision for radical therapy	46 (56)	55 (67)	60 (73)	66 (80)	69 (84)	
Change in treatment plan	36 (44)	27 (33)	22 (27)	16 (20)	13 (16)	*p* = 0.000428
Increase in focal therapy ablation zone	28 (35)	21 (26)	18 (22)	13 (16)	10 (12)	
*Increase by 1 quadrant*	25	20	17	12	10	
*Increase by* ≥*2 quadrants*	3	1	1	1	0	
Unsuitable for focal therapy (ie. recommend radical therapy)	8 (10)	6 (7)	4 (5)	3 (4)	3 (4)	

Compared to the targeted plus reduced-core strategies (Strategy 1–4), a combined targeted and full systematic saturation biopsy resulted in change to the focal therapy treatment plan in 12% (Strategy 1–2/3 core-reduction), 19% (Strategy 2–1/2 core-reduction), 24% (Strategy 3–1/3 core-reduction) and 29% (1/4 cores) of the time (Table 4, *p* = 0.0434). The majority of the changes in treatment plan were contributed by an increase in focal therapy ablation zone by one quadrant in 10%, 15%, 19% and 23% of men from Strategy 1 to 4 (Table 4)

**Table 4:** Proportion of men who had changed in treatment plan when compared to treatment plan based on combined targeted and systematic cores.

	Strategy (1): Targeted + 2/3 Systematic Cores	Strategy (2): Targeted + 1/2 Systematic cores	Strategy (3): Targeted + 1/3 Systematic cores	Strategy (4): Targeted + 1/4 Systematic cores	
No change in focal therapy template, *n* (%)	72 (88)	67 (81)	62 (76)	58 (71)	
Change in management, *n* (%)	10 (12)	15 (19)	20 (24)	24 (29)	*p* = 0.0434
Increase in focal therapy ablation zone, n (%)	8 (10)	12 (15)	15 (19)	19 (23)	
*Increase by 1 quadrant*	8	12	15	18	
*Increase b* ≥*2 quadrants*	0	0	0	1	
Unsuitable for focal therapy (i.e. recommend radical therapy)	2 (2)	3 (4)	5 (6)	5 (6)	

## Discussion

Most studies have performed the systematic biopsy independently of the targeted biopsy with systematic biopsy cores possibly overlapping into the regions of interest identified on mpMRI [1–4]. As one of the challenges in interpreting targeted and systemic biopsies is accounting for the overlap between the two, we assiduously removed systemic cores within the target zone in our analysis of csPCa detection to identify foci that are outside of the MRI lesions. We further evaluated the detection rates of various strategies that reduced number of systematic cores. To determine the clinical impact of systematic biopsy core reduction, we performed focal therapy template planning based on the location of csPCa detected on a full systematic biopsy as well as reduced-core strategies and evaluated if this resulted in a significant change in treatment strategies.

Our findings confirm that the sensitivity of systematic biopsy is largely dependent on the number of systematic cores taken. Systematically reducing the mean number of cores from 21.8 to 14.5, 10.9, 7.3 and 5.4 resulted in a statistically significant decrease in overall csPCa detection on non-targeted systematic biopsy in our entire cohort from 21 to 16%, 13%, 9 and 8% respectively. Previous studies have demonstrated that only as little as 5–16% of csPCa may be missed if systematic biopsy is omitted [1–4]. Our prior study showed that systematic biopsy outside of the target lesion only detected csPCa in 21% of the cohort and that by omitting this biopsy, only 3% of our cohort would have had csPCa missed completely after excluding overlapping systematic biopsy cores into the MRI target zones [7]. In the paradigm of radical whole-gland therapies, a saturation systematic biopsy may have a limited effect in modifying treatment decisions, especially in those who had a prior negative systematic biopsy [7]. With the emerging role of focal therapy, accurate identification of all tumour foci with a thorough prostate interrogation is paramount to ensure optimal patient selection and adequate ablation of all regions with csPCa. Almost half of the men (44%) would have their treatment plan modified when a full systematic biopsy was performed as compared to only having had a targeted biopsy, of which about three-quarters would require an increase in focal ablation zone and the other quarter being deemed unsuitable for focal therapy altogether, either through the identification of high-grade or peri-urethral disease.

Our findings not only strongly reaffirm the role of systematic biopsy in the selection of men for focal therapy, it also examines the importance of the intensity of the systematic biopsy to have sufficient sensitivity to pick up foci of csPCa outside of the MRI-identified target lesions. Through modelling various reduced-cores strategies, our study showed that by reducing the number of cores taken on systematic biopsy resulted in greater proportion requiring a change in their treatment plan as compared to a full systematic biopsy. The proportion of men with a change in treatment plan increased from 12 to 29% when we compared a two-thirds core (strategy 1) and one-quarter core strategy (strategy 4).

It is well accepted that prostate cancer is multi-focal in up to 80% of men [14–16]. Clinically-significant cancer foci in the untreated zones remain a threat to the oncological efficacy of focal therapy as a strategy itself. In a review of focal therapy by Ahdoot and colleagues, out-of-field recurrences were found in 4–49% of men on post-treatment prostate biopsy [17]. These out-of-field recurrences may represent under-detection at initial MRI imaging and subsequent biopsy or the de-novo development of multi-focal disease within the prostate [18], though the former is more likely if the post-treatment biopsy was performed within 6–12 months of focal therapy. Hence, a thorough pre-treatment prostate biopsy may represent an underutilised tool in the selection of patients for focal therapy and our study currently adds critical information in this field.

Our study has demonstrated the downstream clinical impact of reducing the number of cores on systematic biopsy strategy in the formulation of treatment decisions, which may ultimately undermine the long-term oncological efficacy of focal therapy as a whole. Importantly, the intensity of the systematic biopsy may represent a form of selection bias that needs to be addressed when comparing oncological efficacy across clinical trials of the various focal ablation therapies. The most-commonly utilised traditional 12-core systematic biopsy is likely inadequate and we advocate that a saturation biopsy be performed for these men who opt for focal therapy. Based on our study which employed a systematic biopsy with a median of 23 cores for median prostate volume of 43.5 mL, we propose a sampling intensity of at least 1 core for every 2 mL of prostate. Overall, our findings address the Achilles’ heel of MR-guided prostate cancer treatment which is that csPCa exists in MRI-negative regions of the gland. Ensuring that csPCa is not missed and appropriately excluding patients from focal therapy is likely critical to the long-term success of MRI and focal therapy for the treatment of prostate cancer. One strength of our modelling strategy is that it allows for comparative assessment of various reduced core biopsies within the same patient, which is not feasible in-vivo.

Our results have to be interpreted within the context of our study design. Our systematic biopsy did not adhere to any standardised protocol and was largely prostate volume dependent and computer generated. Our reduced-core strategy also assumed that systematic biopsy planning was uniform and that systematic reduction of cores would still result in adequate sampling of the entire prostate. A thorough systematic biopsy may not be well-tolerated in patients who are undergoing transrectal biopsy under local anaesthesia, with its associated risk of sepsis and retention of urine. It is currently unclear whether our non-targeted systematic biopsy cores reflected the gross underestimation of the index lesion or true distant, multi-focal csPCa. Significant cancers detected on non-targeted systematic biopsy may reflect underestimation of the index lesion which various other historadiological correlation studies involving whole-mount prostatectomy specimens have shown [19]. Those who argue in favour of focal therapy point towards whole-mount prostatectomy studies which show that index lesion accounts for about 80% of the prostate tumour burden [20] and hence, the metastatic potential [21]. It is uncertain if these smaller foci of untreated disease outside of the index lesion would ultimately impact the long-term oncological outcomes in a significant manner and further analysis on the intensity of pre-focal therapy prostate biopsy and out-of-field recurrences in focal therapy trials may provide interesting answers. Lastly, the proposed plans for focal therapy were made by a single physician (KJT). While experienced in the area of focal therapy, there are likely to be influences on the focal therapy plan due to personal biases or assumptions. Nonetheless, our study, while non-randomised, retrospective in nature and based on theoretical modelling, is presently the largest study in literature to investigate the impact of core reduction in prostate cancer detection and focal therapy planning in the MRI-fusion era.

## Conclusion

Reducing the number of systematic biopsies when performing an MRI-targeted biopsy leads to reduced detection of csPCa and alters the treatment plans for focal therapy, possibly limiting its oncological efficacy. An intensive systematic saturation biopsy may be required to adequately select patients for focal therapy even with MRI-fusion targeting.

## Supplementary information

Supplementary Table 1

Supplementary Table 2
