# Correction: Reducing the number of systematic biopsy cores in the era of MRI targeted biopsy—implications on clinically-significant prostate cancer detection and relevance to focal therapy planning

**DOI:** 10.1038/s41391-022-00513-w

**Published:** 2022-02-28

**Authors:** Alvin Y. M. Lee, Kenneth Chen, Yu Guang Tan, Han Jie Lee, Vipatsorn Shutchaidat, Stephanie Fook-Chong, Christopher W. S. Cheng, Henry S. S. Ho, John S. P. Yuen, Nye Thane Ngo, Yan Mee Law, Kae Jack Tay

**Affiliations:** 1 Department of Urology, Singapore General Hospital, Singapore, Singapore; 2 Health Services Research Unit, Division of Medicine, Singapore General Hospital, Singapore, Singapore; 3 Department of Urology, Sengkang General Hospital, Singapore, Singapore; 4 Department of Pathology, Singapore General Hospital, Singapore, Singapore; 5 Department of Radiology, Singapore General Hospital, Singapore, Singapore

**Keywords:** Outcomes research, Prostate cancer

Correction to: *Prostate Cancer and Prostatic Diseases* 10.1038/s41391-021-00485-3, published online 14 January 2022

The article “Reducing the number of systematic biopsy cores in the era of MRI targeted biopsy—implications on clinically-significant prostate cancer detection and relevance to focal therapy planning”, written by Alvin Y. M. Lee, Kenneth Chen, Yu Guang Tan, Han Jie Lee, Vipatsorn Shutchaidat, Stephanie Fook-Chong, Christopher W. S. Cheng, Henry S. S. Ho, John S. P. Yuen, Nye Thane Ngo, Yan Mee Law and Kae Jack Tay, was originally published electronically on the publisher’s internet portal on 14. January 2022 without open access. With the author(s)’ decision to opt for Open Choice the copyright of the article changed on 25. January 2022 to © The Author(s) 2022 and the article is forthwith distributed under a Creative Commons Attribution 4.0 International License, which permits use, sharing, adaptation, distribution and reproduction in any medium or format, as long as you give appropriate credit to the original author(s) and the source, provide a link to the Creative Commons licence, and indicate if changes were made.

The images or other third party material in this article are included in the article’s Creative Commons licence, unless indicated otherwise in a credit line to the material. If material is not included in the article’s Creative Commons licence and your intended use is not permitted by statutory regulation or exceeds the permitted use, you will need to obtain permission directly from the copyright holder.

To view a copy of this licence, visit http://creativecommons.org/licenses/by/4.0/.

Open Access funding enabled and organized by Projekt DEAL.

