# Supplementary material for: Reducing the number of systematic biopsy cores in the era of MRI targeted biopsy—implications on clinically-significant prostate cancer detection and relevance to focal therapy planning
Source: Prostate Cancer Prostatic Dis. 2022 Jan 14;25(4):720–6. doi: 10.1038/s41391-021-00485-3 (PMC9705237; doi:10.1038/s41391-021-00485-3)
Supplement: Supplementary file 1 — Supplementary Table 1 [file 41391_2021_485_MOESM1_ESM.docx]

| **Supplementary Table 1.** Strategies to model the detection rate of non-targeted systematic biopsy when their cores are reduced uniformly. | | | |
| --- | --- | --- | --- |
| Strategy number | Reduction Factor | Models within each strategy | Mean number of cores in strategy |
| Full non-targeted systematic biopsy | Not Applicable | Not applicable | 21.8 (±8.6) |
| Strategy (1) | 2/3 the number of systematic cores – by removing every third core | Model (a) – Removal of every third core starting from core 1 (cores 2, 3, 5, 6, 8, …)  Model (b) – Removal of every third core starting from core 2 (cores 1, 3, 4, 6, 7, 9, …)  Model (c) – Removal of every third core starting from core 3 (cores 1, 2, 4, 5, 7, 8, 10, …) | 14.5 (±5.7) |
| Strategy (2) | 1/2 of systematic cores – by taking odd and even cores | Model (a) – Every odd-numbered core starting from core 1 (core 1, 3, 5, …)  Model (b) – Every even-numbered core starting (core 2, 4, 6, …) | 10.9 (±4.3) |
| Strategy (3) | 1/3 the number of systematic cores – by taking every third core | Model (a) – Every third core starting from core 1 (core 1, 4, 7, 10, …)  Model (b) – Every third core starting from core 2 (core 2, 5, 8, 11, …)  Model (c) – Every third core starting from core 3 (core 3, 6, 9, 12, …) | 7.3 (±2.9) |
| Strategy (4) | 1/4 the number of systematic cores – by taking every fourth core. | Model (a) – Every fourth core starting from core 1 (core 1, 5, 9, 13, …)  Model (b) – Every fourth core starting from core 2 (core 2, 6, 10, 14, …)  Model (c) – Every fourth core starting from core 3 (core 3, 7, 11, 15, …)  Model (d) – Every fourth core starting from core 4 (core 4, 8, 12, 16, …) | 5.4 (±2.2) |
