# Supplementary material for: Reducing the number of systematic biopsy cores in the era of MRI targeted biopsy—implications on clinically-significant prostate cancer detection and relevance to focal therapy planning
Source: Prostate Cancer Prostatic Dis. 2022 Jan 14;25(4):720–6. doi: 10.1038/s41391-021-00485-3 (PMC9705237; doi:10.1038/s41391-021-00485-3)
Supplement: Supplementary file 2 — Supplementary Table 2 [file 41391_2021_485_MOESM2_ESM.docx]

| **Supplementary Table 2.** Distribution of focal therapy templates / treatment recommendations depending on pattern of clinically-significant cancers detected by the various biopsy strategies (n=82) | | | | | | |
| --- | --- | --- | --- | --- | --- | --- |
|  | All cores (Targeted + all systematic cores) | Targeted cores only | Strategy (1):  Targeted + 2/3 Systematic Cores | Strategy (2): Targeted + 1/2 Systematic cores | Strategy (3):  Targeted + 1/3 Systematic cores | Strategy (4):  Targeted + 1/4 Systematic cores |
| Single quadrant ablation, n (%) | 18 (22) | 26 (32) | 22 (27) | 22 (27) | 23 (28) | 24 (29) |
| Hemi-ablation, n (%) | 18 (22) | 14 (17) | 16 (20) | 17 (20) | 16 (20) | 15 (18) |
| 2-quadrant discontinuous ablation, n (%) | 6 (7) | 3 (4) | 5 (6) | 4 (5) | 3 (4) | 3 (4) |
| 3-quadrant ablation, n (%) | 5 (6) | 2 (2) | 4 (5) | 3 (4) | 4 (5) | 3 (4) |
| Whole-gland ablation, n (%) | 1 (1) | 0 | 1 (1) | 1 (1) | 1 (1) | 1 (1) |
| Whole-gland Radical therapy (RP or RT), n (%) | 34 (42) | 26 (32) | 32 (39) | 31 (38) | 29 (35) | 29 (35) |
| Active surveillance (conservative therapy, n (%) | 0 | 11 (13) | 2 (2) | 4 (5) | 6 (7) | 7 (9) |
